# Supplementary material for: DMRforPairs: identifying Differentially Methylated Regions between unique samples using array based methylation profiles
Source: BMC Bioinformatics. 2014 May 15;15:141. doi: 10.1186/1471-2105-15-141 (PMC4046028; doi:10.1186/1471-2105-15-141)
Supplement: Additional file 2 — DMRforPairs output for the comparison of A431-MCF7 and NA17018-NA17105. Please start from the HTML files in each folder. Available via the BMC Bioinformatics website. [file 1471-2105-15-141-S2.zip › 1394847754114233_MOESM2_ESM/A431_MCF7/figures/14972.pdf]

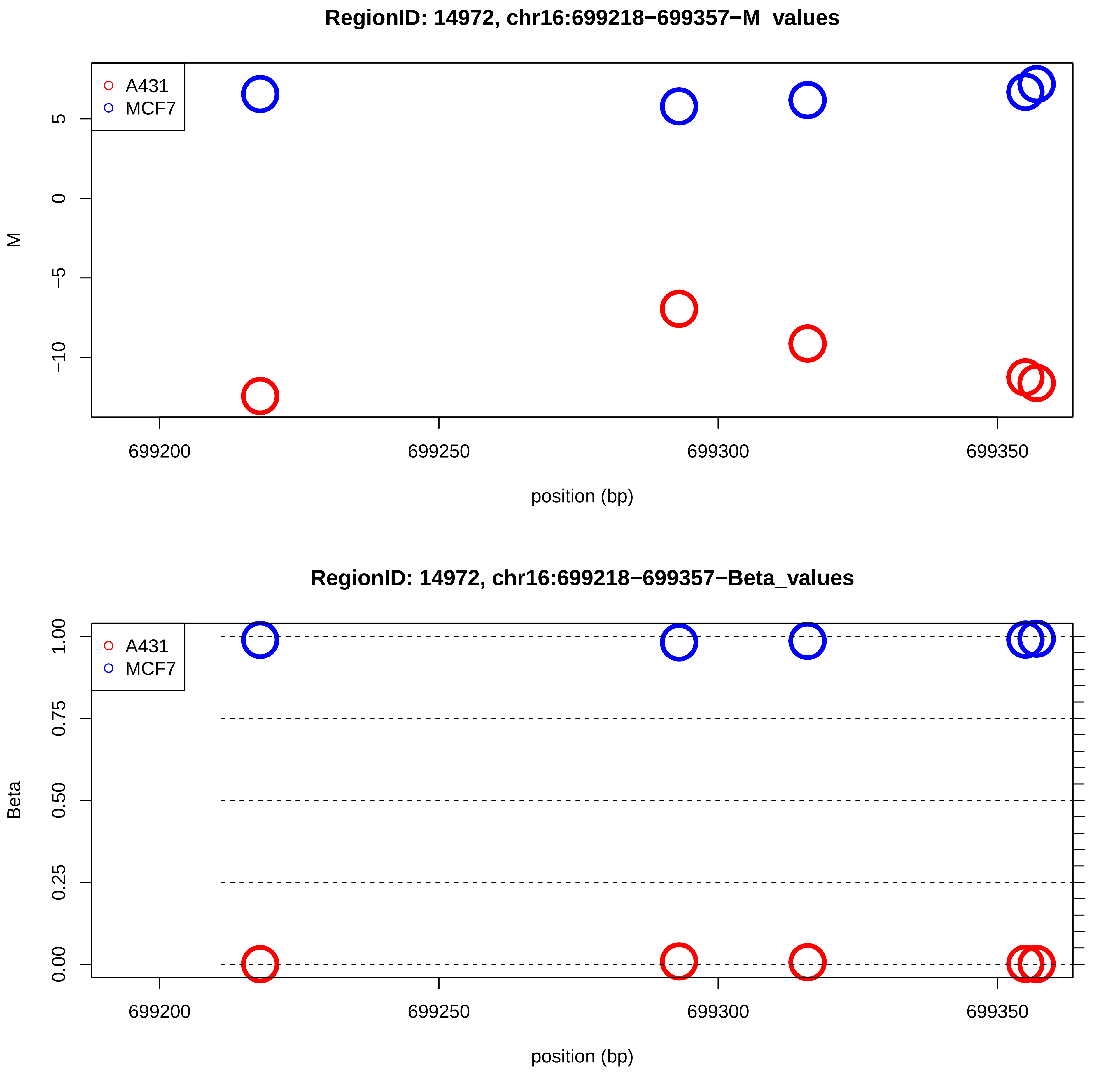
RegionID: 14972, chr16:699218−699357−M_values
A431
MCF7
M
5
0
−5
−10
699200
699250
699300
699350
position (bp)
RegionID: 14972, chr16:699218−699357−Beta_values
A431
MCF7
Beta
1.00
0.75
0.50
0.25
0.00
699200
699250
699300
699350
position (bp)

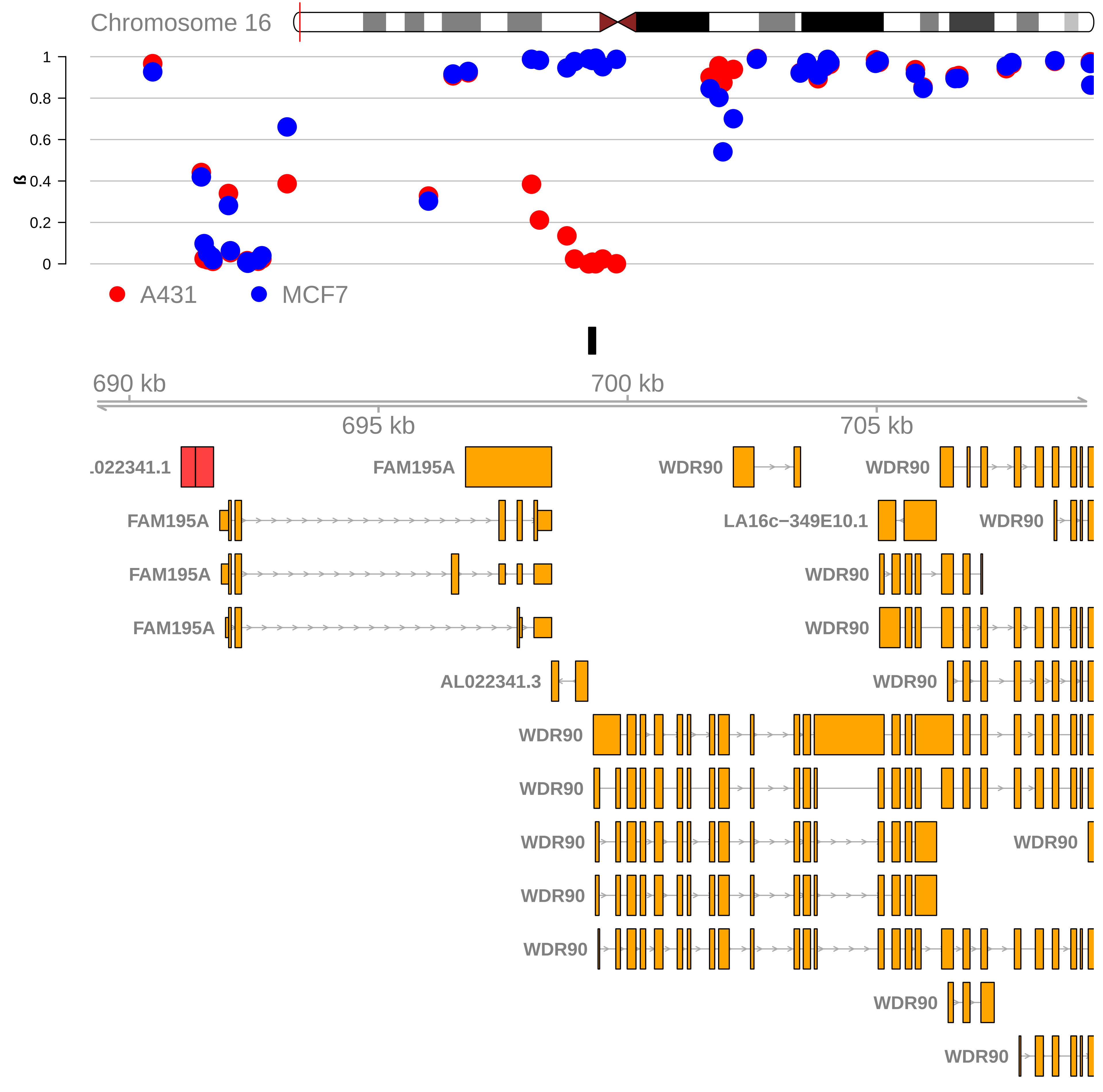

Chromosome 16
β
1
0.8
0.6
0.4
0.2
0
A431
MCF7
690 kb
695 kb
700 kb
705 kb
.022341.1
FAM195A
WDR90
LA16c−349E10.1
AL022341.3
